# ‘Primary’ antibiotics in wastewater treatment plants

**DOI:** 10.1016/j.isci.2024.110789

**Published:** 2024-08-22

**Authors:** Sangeeta Adhikari, Hong H. Lee, Do-Heyoung Kim

**Affiliations:** 1 School of Chemical Engineering, Chonnam National University, 77 Yongbong-ro, Gwangju 61186, Republic of Korea; 2 School of Chemical and Biological Engineering, Seoul National University, Gwanak-ro, Seoul 08826, Republic of Korea

**Keywords:** Environmental science, Pollution, Biological waste treatment, Water resources engineering

## Abstract

There are anywhere from 5 to 8 priority antibiotics in typical wastewater treatment plants (WWTPs) whose concentrations exceed the maximum allowed, out of 12 priority antibiotics designated by the World Health Organization as the species to pose severe health hazard than others. If the priority antibiotics to deal with could be reduced to just one or two, such reduction would greatly simplify the construction and operation of the treatment plants. Introduced here is a concept of ‘primary’ antibiotic, the abatement of which ensures mitigation of all the other priority antibiotics in the wastewater. A criterion for determining primary antibiotic is developed. For a demonstration of the approach, the wastewater systems treated with solar-based photocatalysts are considered. The criterion reveals that the primary antibiotic in the typical European WWTP as well as in the typical municipal and hospital wastewater is ciprofloxacin, whereas the typical industrial wastewater contains ciprofloxacin and oxytetracycline.

## Graphical abstract

**Figure:**
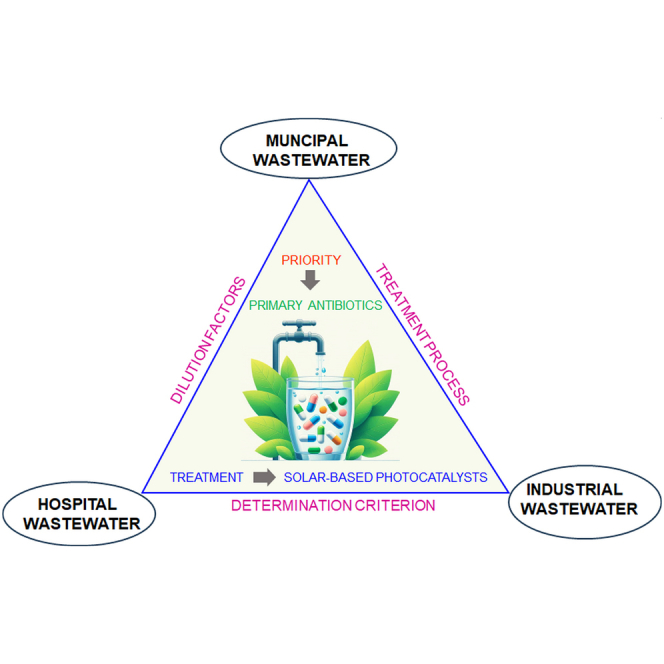

## Introduction

Antibiotics have been wonder drugs since the introduction of penicillin in the early 20th century. Since then, antibiotics have evolved, and they have been used not only for human beings but also for food animals and even as growth promoters in aquaculture and agriculture.^1^^,^^2^^,^^3^^,^^4^ As such, the amount of antibiotics used worldwide is ever increasing, exceeding 100,000 tons/yr.^5^ However, the antibiotics are not completely metabolized when taken by human beings and food animals.^2^ For instance, approximately 90% of the antibiotics consumed by animals are excreted^6^ in the original or modified form, eventually entering wastewater treatment plants (WWTPs).^2^ This continuous emission of large amounts of antibiotics into the environment promotes antibiotic resistance in bacterial populations.^7^^,^^8^ Antibiotic resistant organisms also enter into water environments from human and animal sources.^9^ Antibiotic resistant bacteria (ARB) and genes (ARG) have become a real threat to human health.^10^ According to the 2015 data, ARB caused more than 100,000 deaths and this number was expected to rise with time,^11^^,^^12^ possibly reaching millions annually by 2050.^13^

There are two general approaches for dealing with antibiotics: physical collection^14^ and chemical transformation.^15^^,^^16^ Membrane separation, adsorption, and other physical means of removal belong to the former category. Physical collection simply moves the antibiotics from one place to another and the collected antibiotics have to be treated in some way, either burned or buried. There could be potential risks in handling the post-collected samples. In contrast, no post-treatment is needed in the chemical transformation or degradation case.

A number of methods are available for the chemical transformation of antibiotics. Of these, advanced oxidation processes (AOP) are touted as the most efficient remediation route.^17^^,^^18^^,^^19^ Depending on how the reactive oxygen species (ROS) are generated, they are grouped into electrochemical, ionizing irradiation, photochemical, sonochemical, Fenton-photo oxidation, and others. In these AOPs, catalysts are used to provide efficiency in the treatment of the antibiotics.

There are numerous antibiotics present in wastewater. In a study covering 48 WWTPs from 11 countries in Europe,^20^ for instance, 47 antibiotics were found. In another study, more than 70 antibiotics were detected in 7 major water systems in China.^21^ In face of the large number of antibiotics present, the WWTPs have a number of challenging issues to resolve. These include the number of different catalysts needed for the many antibiotics present, the sequence of treatments, and the size of the treatment reactors.

In this work, the concept of ‘primary’ antibiotics is introduced to resolve the challenging issues discussed above at the WWTPs. A criterion is derived that can be used to determine these primary antibiotics. The approach taken, which is applicable to other antibiotics treatment processes, is demonstrated for the solar-based photocatalytic treatment of the antibiotics in typical WWTPs. In the demonstration, primary antibiotics are also identified for families of antibiotics.

### Priority antibiotics and treatment requirement

Numerous antibiotics are present in wastewater. It is, therefore, prudent to choose and focus on those antibiotics that are more hazardous than the others. It is of interest in this regard that there were 12 antibiotics that the World Health Organization (WHO) listed as priority antibiotics.^22^ On the basis of the data on 631 different pharmaceuticals in 20 different environmental matrices across 71 countries, Booth et al.^23^ provided the mean concentrations of these 12 antibiotics present in various wastewater sources, which is reproduced in Table 1 with the concentrations rounded to larger numbers. It is notable that out of these 12 priority antibiotics, only some of them have the mean concentrations exceeding the predicted no-effect concentration (PNEC),^20^ below which the antimicrobial resistance is not likely to develop: 5 out of 12 in the municipal wastewater, 6 in the hospital wastewater, and 7 in the industrial wastewater.

**Table 1:** Mean concentrations (μg/L) of 12 priority antibiotics and 2 high risk antibiotics

Antibiotic	PNEC	Type of Wastewater			Risk Score^a^
		Municipal	Hospital	Industrial	
Amoxicillin	0.250				
Azithromycin	0.250		1.0		1.89
Ciprofloxacin	0.064	600	7.0	3600	1.09
Clarithromycin	0.064	0.2	3.0	0.1	1.43
Clindamycin	1.000				
Doxycycline	2.000				
Enrofloxacin	0.064	60	20	30	
Ofloxacin	0.500	4.0	5.0		2.85
Oxytetracycline	0.500			24000	
Sulfamethoxazole	16.000			19000	
Tetracycline	1.000			500	
Trimethoprim	0.500	1.0	2.0	3100	
Erythromycin	1.0^b^				1.66
Cefalexin	0.08^b^				

a Antibiotics with risk score higher than 1.0 were classified as priority antibiotics in Zhao and Xiao; Ng et al.^20^^,^^24^

b High risk antibiotics taken from Wang and Zhuan; Rodriguez-Mozaz et al.^17^^,^^25^

**Figure 1:**
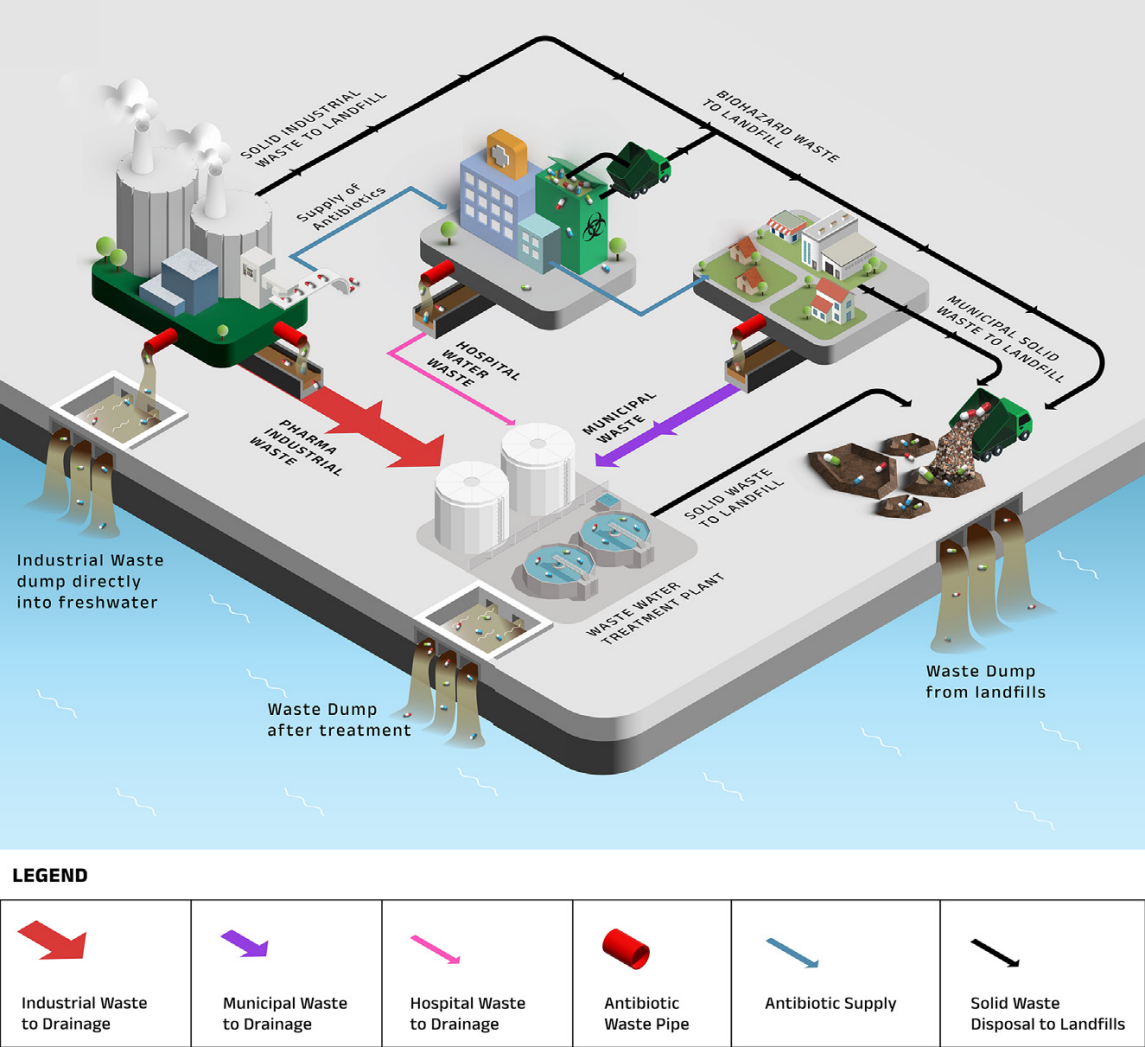
Release of wastewater containing antibiotics from different sectors, probable dilutions (thicker arrows demonstrate the least dilution) and mixing pathways in the ecological system

The wastewater treatment plant has to reduce the level of antibiotics below PNEC. This PNEC requirement is further relaxed by what is known as dilution factor (DF),^25^ which is the ratio of river flow rate to wastewater flow rate. Since the effluents from the treatment plants are diluted as they are mixed with river water, the treatment requirement of the antibiotics is in effect the PNEC multiplied by the DF, raising the permitted outlet concentration of the plant as depicted in Figure 1. Therefore, the outlet concentration that has to be reached after the treatment, or the treatment requirement, is (PNEC)(DF). The DF can vary from near 1 to over 1000 depending on the location, the variation being different even in the same country.^26^ The usual engineering practice^27^ is to use a DF of 40.

Also shown in the table are the priority antibiotics chosen in a study on the effluents from 48 WWTPs in 11 countries of Europe.^20^ A risk score was developed to assess the risk posed by the antibiotics and those with the score higher than 1.0 were designated as priority antibiotics. These antibiotics are seen to overlap with the priority antibiotics that were classified by the WHO except for erythromycin, as shown in the table.

There were also other extensive studies on the antibiotics in wastewater.^28^ In a study on the antibiotics in 13 WWTPs in 7 countries of Europe, cefalexin, ciprofloxacin, and azithromycin were identified as the antibiotics that pose moderate risk to the environment.^25^ In another extensive study on the antibiotics from the WWTPs all across China,^29^ the top 5 antibiotic residues in the effluents were cefalexin, amoxicillin, erythromycin, ofloxacin, and trimethoprim. These antibiotics also coincide with the priority antibiotics listed in Table 1, if erythromycin and cefalexin are included. Therefore, these 14 antibiotics may be taken as priority antibiotics for further consideration.

Although local conditions could prevail to change the priority antibiotics, those listed in Table 1 provide a good reference for preparing test solutions of antibiotics. In terms of chemical structure, there are 4 macrolides (azithromycin, clarithromycin, clindamycin, and erythromycin) out of the 14 priority antibiotics, 3 fluoroquinolones (ciprofloxacin, enrofloxacin, and ofloxacin), 3 tetracyclines (doxycycline, oxytetracycline, and tetracycline), 2 beta-lactams (amoxicillin, and cefalexin), 1 sulfonamide (sulfamethoxazole), and 1 diaminopyrimidine (trimethoprim).

The municipal wastewater in Table 1 has 5 antibiotics whose measured concentrations exceed the PNECs. They are: 3 fluoroquinolones (ciprofloxacin, enrofloxacin, and ofloxacin), 1 macrolide (clarithromycin), and 1 diaminopyrimidine (trimethoprim). The most severe burden on the catalyst falls on ciprofloxacin, for which the catalyst has to decompose or degrade the antibiotic from 600 to below 0.064 μg/L. Even if the DF of 100 is used, it needs to reduce the concentration from 600 to 6.4 μg/L (0.064 × 100). Even for this more lenient DF of 100, the required conversion or degradation level is almost 99%. The conversion required of the other two fluoroquinolones is much lower than that of ciprofloxacin. Considering that the catalyst effective for ciprofloxacin could also be good for the other two fluoroquinolones because of similar chemical structure, the conversion levels could be of minor issue.

With the DF of 100, the dilution itself is sufficient to bring the concentrations of the other 2 priority antibiotics, clarithromycin and trimethoprim, below the PNECs, the resultant concentrations being 0.002 and 0.01 μg/L. In consequence, no additional catalyst may be needed. Therefore, it is possible that one catalyst could be sufficient for the treatment of the municipal wastewater.

In contrast to municipal wastewater treatment, the industrial wastewater treatment in Table 1 may require a combination of catalysts, as DF of 100 will not be able to bring down the concentration below PNEC, except for clarithromycin. Furthermore, the burden on the catalysts is really high because of the high antibiotics’ concentration. The conversion required of ciprofloxacin is 99.998% even with the DF of 100 to bring the concentration down below the PNEC. The conversion required of oxytetracycline is also 99.998% and that of trimethoprim is 99.984%. These levels of conversion take quite a long reaction time. The high level of conversion and the diversity of catalysts needed for 5 different chemical structures of fluoroquinolone, macrolide, tetracycline, sulfonamide, and diaminopyrimidine would pose a great challenge for any catalyst system that is designed to treat the industrial wastewater.

In Table 1, risk scores given in the sixth column serve as an indicator for prioritization of the high-risk antibiotics found in the ecosystem. To elaborate, it is the summation of frequency of appearance, PNEC exceedance and extent of PNEC exceedance that are scaled from 0 to 1 for each. A detailed comprehension could be found elsewhere along with prioritization scheme.^24^^,^^30^ The risk score obtained, indicating values exceeding 1, designates antibiotics of high priority. This suggests the potential necessity of employing a combination of catalysts for such antibiotics in WWTPs, particularly those containing fluoroquinolones (FQs) like ofloxacin and ciprofloxacin, as well as macrolides (MLs) such as erythromycin. This need for multiple catalysts is similarly observed in WWTPs in China, where high-priority antibiotics include ofloxacin, erythromycin, and trimethoprim.^29^

### ‘Primary’ antibiotics in typical European WWTP wastewater

The sixth column in Table 1 lists the priority antibiotics that were judged to pose a risk to environment on the basis of the evaluation of 676 antibiotics found in 48 WWTPs in 11 European countries. As such, the column can be considered to represent typical European WWTP wastewater. Out of the 14 priority antibiotics, the wastewater contains 2 FQs of ciprofloxacin (CIP) and ofloxacin, and 3 MLs of azithromycin, clarithromycin, and erythromycin.

With numerous antibiotics present in the wastewater, it would greatly simplify the construction and operation of the antibiotics treatment facilities if one could focus only on a minimum number of antibiotics, or even one. For this to be true, the abatement of these minimum antibiotics should guarantee the mitigation of the other antibiotics, and as such these antibiotics may be termed ‘primary’ antibiotics.

In most studies on the degradation of antibiotics, the degradation has been modeled as a pseudo-first order reaction. For a first order reaction taking place in a plug flow reactor, the outlet concentration, C_o_, is given by C_o_ = C_i_ exp (-kt_r_) where C_i_ is the inlet concentration of the antibiotic, k is the rate constant, and t_r_ is the residence time or reaction time. The residence time is the reactor volume divided by the feed flow rate and thus for a given throughput, t_r_ represents the reactor size. For the treatment reactor, the outlet concentration must be below the PNEC of the antibiotic of interest. Because of the dilution effect afforded by the DF, the outlet concentration can be relaxed to (PNEC)(DF), effectively raising the desired outlet concentration by the factor of (DF). Then the conversion relationship can be rearranged as follows for the residence time:

(Equation 1) $$t_{r}=-\;\left(1/k\right)\ln\left[\left(\text{PNEC}\right)\left(\text{DF}\right)/\left(C_{i}\right)\right]$$

This residence time may be taken as a measure for determining the primary antibiotics. As indicated earlier, the residence time is equivalent to the size of the treatment reactor for a given throughput. If the residence time of a particular antibiotic is larger than those of the other antibiotics in the wastewater, all it takes for the treatment of all the antibiotics in the wastewater is to bring the concentration of that antibiotic below the desired level, since then the other antibiotics will be reacted longer than the required residence times for the species, leading to the concentrations below their PNECs.

Residence time, beyond its quantitative significance, holds sway over both capital expenditure (Capex) and operating costs. Additionally, they can impact by accumulation and disturbing the habitat integrity. Antibiotics necessitating longer residence times may demand robust, sizable reactors. Enlarged equipment, including mixers, pumps, and aeration systems, escalates Capex requirements. Moreover, energy consumption for equipment operation and reactor functioning, coupled with potential maintenance needs, adds to operating expenses. While prolonged residence time can foster complete chemical reactions with reduced catalyst dosages, shorter residence times might mandate higher catalyst quantities. Consequently, residence time correlates with the comprehensive operational cost in WWTPs.

In the body of the wastewater being considered, the concentration of azithromycin (AZI) and that of clarithromycin (CLA) are higher than that of erythromycin (ERY).^31^ In a study^32^ on the degradation of antibiotics with TiO_2_ as a catalyst and Na_3_S_2_O_8_ as an oxidant under sunlight, the pseudo-first order rate constants were about the same for CLA and ERY (Table 2). The rate constant was 0.966 min^−1^ for CLA and 0.942 for ERY in pure water whereas it was 0.043 min^−1^ for CLA and 0.053 for ERY in wastewater. The decrease in the rate constant by an order of magnitude was attributed^32^ to the occupancy of the active sites on TiO_2_ by organic and inorganic components and to the scavenging of reactive oxygen species (ROS) by the ions in the wastewater. Therefore, either CLA or AZI must be the primary ML out of the MLs in the typical European WWTP wastewater.

**Table 2:** List of priority antibiotics treated with different photocatalysts along with their reaction parameters such as initial concentration, rate constants and degradation percentage

Antibiotics	Photocatalyst	Reaction Time	Light source	Initial Concentration of Antibiotics	Rate constant (min^−1^)	Degradation (%)	Reference
Clarithromycin	TiO_2_	240 min	UV light	100μg/L	0.9656 (Pure water) 0.043 (Wastewater)	–	Pérez-Lucas et al.^32^
Erythromycin				100μg/L	0.9421 (Pure water) 0.053 (Wastewater)	–	
Azithromycin	Gd^3+^ doped BiVO_4_	180 min	UVA-LED	892 ng/L	–	62.9% (wastewater)	Orona-Návar et al.^33^
Clarithromycin				196 ng/L	–	27.5% (wastewater)	
Ciprofloxacin				9687 ng/L	–	30% (wastewater)	
Erythromycin	TiO_2_	66 min	Sunlight	180 ng/L	–	99% (wastewater)	Rueda-Márquez et al.^34^
Ciprofloxacin				3310 ng/L	–	93% (wastewater)	
Ofloxacin				156 ng/L	–	84% (wastewater)	
Azithromycin				250 ng/L	–	86% (wastewater)	
Ciprofloxacin	g-C_3_N_4_	100 min	Simulated sunlight source	4 mg/L	0.042 (Pure water)	92.3% (Pure water)	Wang et al.^35^
Enrofloxacin				4 mg/L	0.06 (Pure water)	–	
Ofloxacin				4 mg/L	0.045 (Pure water)	–	
Ciprofloxacin	TiO_2_-N	40 min	UV_solar_	100 μg/L	0.11 (Pure water)	93%	Venancio et al.^36^
Ofloxacin				100 μg/L	0.08 (Pure water)	83%	
Enrofloxacin	TiO_2_	60 min	UV _(λ>324nm)_	100 μM	0.119 (Pure water)	–	Paul et al.^37^
Ciprofloxacin				100 μM	0.0137 (Pure water)	–	
Tetracycline	BiVO_4_/TiO_2_/rGO	120 min	1000 W Xe-lamp _(λ>420nm)_	10 μg/mL	0.0261 (Pure water)	96.2%	Wang et al.^38^
Oxytetracycline				10 μg/mL	0.0288 (Pure water)	98.7%	
Tetracycline	BiVO_4_	120 min	Solar light	10 mg/L	0.0102 (Pure water)	93%	Hemavibool et al.^39^
Oxytetracycline				10 mg/L	0.0093 (Pure water)	72%	
Tetracycline	MoSSe	60 min	Visible light	10 mg/L	0.10 (Pure water)	95%	Chen et al.^40^
Oxytetracycline				10 mg/L	0.08 (Pure water)		
Ciprofloxacin	TiO_2_/WO_3_	120 min	Natural Sunlight	20 mg/L	0.034 (Pure water)	96%	Moghni et al.^41^
Oxytetracycline				20 mg/L	0.028 (Pure water)	97%	
Ciprofloxacin	WO_3_-Carbon nanodots		Solar simulator	10 mg/L	0.00154 (Pure water)	62.5%	Zwane et al.^42^
Sulfamethoxazole				10 mg/L	0.00359 (Pure water)	60%	

In the treatment of an urban wastewater^33^ containing 892 ng/L of AZI and 196 ng/L of CLA among other antibiotics, the BiVO_4_ catalyst doped with Gd degraded AZI by 60% when the wastewater effluent was irradiated with UVA for 280 min whereas it degraded CLA by 27%. In another study^31^ involving yet another urban wastewater, the degradation efficiency was almost the same for CLA and AZI when the degradation was catalyzed by a TiO_2_ catalyst for the effluent containing approximately 30% more AZI than CLA. Thus, the rate of degradation of CLA is lower than or approximately the same as that of AZI. Furthermore, the PNEC of CLA is much lower than that of AZI (0.064 vs. 0.250 μg/L), as shown in Table 1. Therefore, the primary ML for the wastewater can be taken to be CLA.

The typical European WWTP wastewater being considered contains not only 3 MLs but also 2 FQs of CIP and ofloxacin, as shown in Table 1. As shown shortly, CIP is the primary antibiotic among FQs. Therefore, the presence of the primary FQ of CIP in the wastewater raises a natural question as to which of the two, CLA and CIP, could be the primary antibiotic of the WWTP wastewater.

In the study by Orona-Navar^33^ for CLA, CIP was also included, for which the initial concentration was 9687 ng/L and the same catalyst degraded it by 30%. Thus, the rate of degradation of CIP was about the same as that of CLA (30 vs. 27%) but the concentration of CIP was larger by more than an order of magnitude (196 vs. 9687 ng/L). Therefore, the residence time given by Equation 1 is larger for CIP than for CLA because the PNECs for both are the same at 0.065 μg/L (Table 1). Hence CIP could be the primary antibiotic for wastewater.

Another study^34^ involving an urban WWTP effluent adds credence to CIP being the primary antibiotic for the wastewater. The effluent had, among various antibiotics, 180 ng/L of ERY and 3310 ng/L of CIP. When the effluent was treated with a TiO_2_ catalyst under sunlight for 66 min, ERY was degraded by 99% and CIP by 93%, which indicates that the rate of degradation of CIP is smaller than that of ERY. Noting that the rate constant for ERY was about the same as that of CLA for a TiO_2_ catalyst, which was discussed earlier,^32^ it could be construed that the same conclusion would apply to CLA. Application of Equation 1 should indicate that CIP is the primary antibiotic.

It should be pointed out that the local conditions of WWTP could be different from those of the typical European WWTP wastewater considered here and therefore, the conclusion on the primary antibiotic could be different depending on the local conditions.

**Figure 2:**
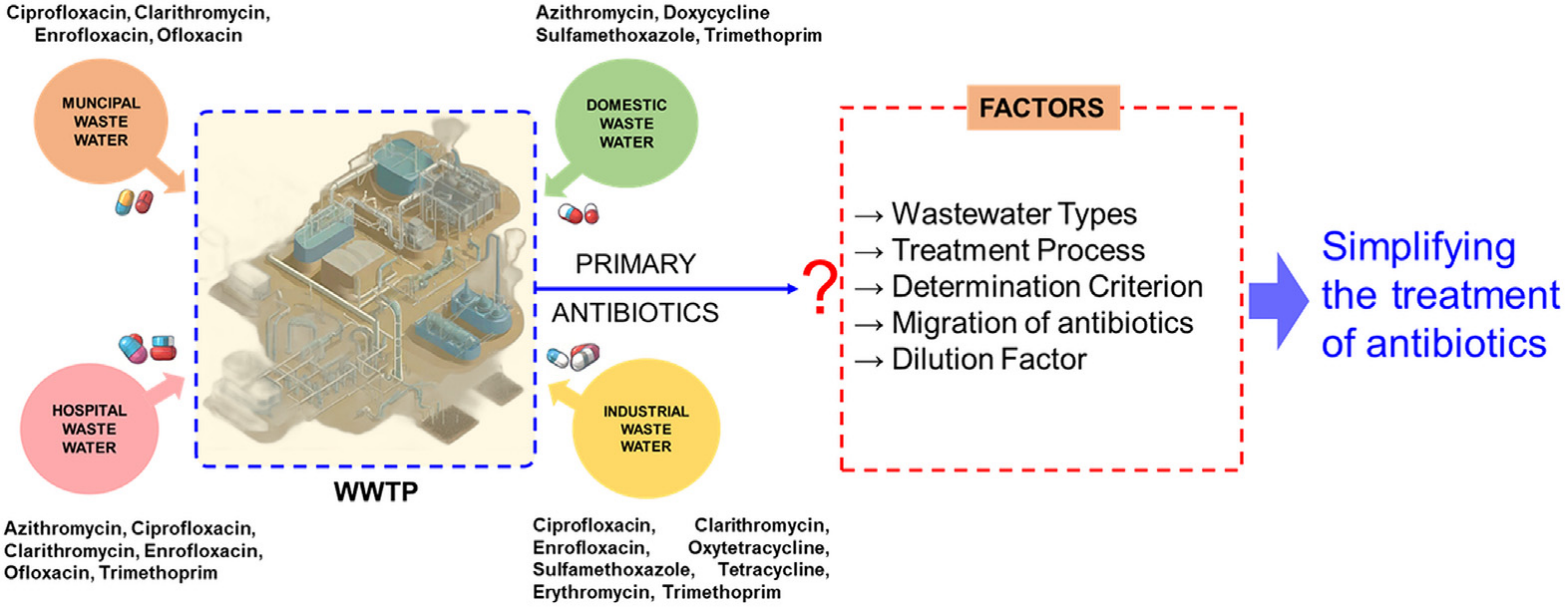
The major antibiotics identified from different sectors and factors governing to simplify the treatment

The definition of the primary antibiotic and its utilization should make it highly valuable for the construction and operation of WWTPs. Instead of paying attention to all the antibiotics present in the wastewater, it allows one to focus only on the primary antibiotic, the abatement of which ensuring control of all the other antibiotics in the wastewater through factors regulator (Figure 2).

### Primary antibiotics in typical municipal and hospital wastewater

The municipal wastewater represented by the third column in Table 1 may be taken as a typical municipal wastewater in that it was constructed on the basis of the data on 631 different pharmaceuticals in 20 different environmental matrices across 71 countries. Although there are ML, diaminopyrimidine, and FQs whose concentrations exceed the corresponding PNECs, only the FQs need to be treated, considering the usual DF. This fact suggests that only one catalyst could be sufficient for the treatment of the typical municipal wastewater. The same should apply to the hospital wastewater shown in the fourth column of Table 1 because it contains similar priority antibiotics at concentrations lower than those in the municipal wastewater. The control over the FQs is of particular interest in that they are the only antibiotics that are present with the concentrations exceeding their PNECs in surface water (river and stream).^23^ In particular, the concentration of ciprofloxacin (CIP) far exceeds its PNEC (384 vs. PNEC of 0.064 μg/L).

Among the primary antibiotics present in the typical municipal wastewater in Table 1, all the antibiotics except for CIP and enrofloxacin (ENR) would meet the PNEC requirements if a DF of 40 were used, a DF usually taken for engineering practice. The question is then which of the two would be the primary antibiotic.

It can be speculated that a catalyst effective for CIP could also be efficient for the other FQs because of the similarity in chemical structure. In fact, Wang et al.^35^ found for a mesoporous graphitic carbon nitride (g-C_3_N_4_) catalyst that the pseudo-first order rate constant for ENR was 0.06 min^−1^, whereas those for CIP and ofloxacin were similar at 0.042 min^−1^. Venancio et al.^36^ determined that a TiO_2_-N catalyst impregnated with urea yielded a rate constant of 0.11 min^−1^ for CIP and 0.08 min^−1^ for ofloxacin. In a detailed study on the degradation of FQs for a TiO_2_ catalyst,^37^ the rate constant determined for ENR was 0.119 min^−1^ and that of CIP 0.0135 min^−1^ under identical reaction conditions.

As shown in Table 1, the PNECs for both CIP and ENR are the same at 0.064 μg/L whereas the concentration of CIP in the wastewater, which is the inlet concentration to a treatment reactor, is an order of magnitude larger than that of ENR. Therefore, the residence time given by Equation 1 is larger for the treatment of CIP than that for ENR, given the fact that the rate constant for CIP is smaller than that for ENR. Thus, CIP is the primary antibiotic for the municipal wastewater according to the criterion provided by the equation.

For the typical hospital wastewater given in Table 1, only CIP and ENR need to be considered for the determination of the primary antibiotic if we apply the same argument as that for the municipal wastewater. Application of Equation 1 should reveal that CIP is the primary antibiotic, although the value for ENR is not much smaller than that for CIP. It can be concluded, therefore, that the primary antibiotic is CIP for both the municipal and hospital wastewater.

### Primary antibiotics in typical industrial wastewater

The typical industrial wastewater represented by the fifth column of Table 1 has 2 FQs, 1 ML, 2 tetracyclines (TCs), 1 sulfonamide, and 1 diaminopyrimidine. Out of the 14 priority antibiotics, these are the priority antibiotics whose concentrations exceed their PNECs. The wastewater is typified by the relatively huge concentrations of priority antibiotics compared with the municipal or hospital wastewater.

It is obvious from the antibiotic concentrations and the PNECs shown in the table that the oxytetracycline (OXY) would be the primary TC for this wastewater unless the rates of degradation make a difference. The pseudo-first order rate constants of tetracycline (TET) and OXY, when the degradation was catalyzed by a composite of BiVO_4_/TiO_2_/rGO under visible light, were 0.0261 and 0.0288 min^−1^, respectively.^38^ With BiVO_4_ as the catalyst, solar light irradiation yielded rate constants of 0.0102 and 0.0093 min^−1^, respectively, for TET and OXY.^39^ On the other hand, visible light irradiation with a MoSSe nanohybrid produced rate constants of 0.10 and 0.08 min^−1^, respectively, for TET and OXY.^40^ These results clearly show that the rate constants of TET and OXY are of the same order of magnitude. Therefore, OXY can be concluded to be the primary TC because of the concentration of OXY that is much higher than that of TET in the wastewater.

However, there still remains the question as to which antibiotics of CIP and OXY could be the primary antibiotic for industrial wastewater. The wastewater concentrations and PNECs for these primary FQ and TC in Table 1 provide the values of the logarithm in Equation 1 to be - 6.33 and - 6.18 for a DF of 100, respectively, for the primary FQ of CIP and the primary TC of OXY. A recent study^41^ compared the degradation behavior of these two antibiotics under sunlight with a catalyst of TiO_2_/WO_3_. It turned out that the rate of CIP degradation was somewhat faster than that of OXY, meaning that the rate constant for CIP is larger than that of OXY. A similar result was reported in another study^43^ involving a polyoxometalate/polymer composite catalyst. These results indicate that the residence time given by Equation 1 for CIP should be similar to that of OXY. Therefore, CIP is also a primary antibiotic for industrial wastewater.

The conclusion on CIP and OXY still leaves sulfamethoxazole (SUL) and trimethoprim (TRI) for further consideration. A study^42^ on the degradation of CIP and SUL found SUL was degraded faster than CIP by a composite catalyst of WO_3_ and carbon nanodots when irradiated by a simulated sunlight. Therefore, SUL cannot be a primary antibiotic of the industrial wastewater according to Equation 1.

The degradation efficiency of TRI was slightly lower than that of CIP, approximately around 30%, when they were treated with a doped BiVO_4_ catalyst under an LED-UVA light,^33^ indicating that the rate constant for CIP is somewhat higher than that of TRI. On the other hand, the concentration remaining after 1 h exposure to sunlight was 0.088 and 0.050 mol/g for CIP and TRI, respectively, when they were treated with a TiO_2_ catalyst.^44^ For both, the same 0.2 M solutions were used. This result shows that the rate constant for CIP is lower than that of TRI. It is, therefore, difficult to take TRI as a primary antibiotic for industrial wastewater.

The factors that determine the primary nature of antibiotics are the concentration of the antibiotic in the wastewater, its PNEC, and the rate constant, as apparent from Equation 1. The first two factors are inherent to a given wastewater and cannot be manipulated. The other factor of rate constant, however, can be made larger by improving the catalyst performance. Any improvement in the catalytic performance is bound to be felt the same way by all the members of the family due to the same basic chemical structure. Therefore, designation of the primary antibiotic for a given family of antibiotics will endure the test of time. Examples are CIP for fluoroquinolones, CLA for macrolides, and OXY for tetracyclines. On the other hand, the designation of primary antibiotic for a given wastewater could change with time because the improvement in the catalyst may vary from one family to another.

The typical industrial wastewater considered here has been found to contain two primary antibiotics of CIP and OXY. This fact immediately suggests that two catalytic systems would be needed for the wastewater treatment because the catalyst optimal for the primary FQ of CIP may not be optimal for the primary TC of OXY. A physical mixture of two catalysts could be used for this purpose. When the catalysts are immobilized on a support, the two catalysts on the same support may be used, or a physical mixture of two separately supported catalysts could be preferred.

### Concluding remarks

The concept of primary antibiotic has been introduced that enables one to convert a many species problem to a much simpler one or two species problem for WWTPs. A criterion has been derived that can be used to determine the primary antibiotic for a given treatment system. The criterion has been applied to various WWTPs for solar-based photocatalytic systems to demonstrate its utility. Ciprofloxacin has been identified as the primary antibiotic in the typical European WWTP as well as in the typical municipal and hospital wastewater, where there are anywhere from 5 to 8 priority antibiotics present. Both ciprofloxacin and oxytetracycline have been found to be the primary antibiotics for typical industrial wastewater. Abatement of the primary antibiotics automatically ensures mitigation of all the other antibiotics in the wastewater. The approach and criterion developed here are applicable to any given catalyst system for a WWTP. In terms of families of antibiotics, the primary antibiotic for fluoroquinolones is ciprofloxacin, that for the macrolides is clarithromycin, and for tetracyclines, it is oxytetracycline.

### Limitations of the study

From the perspective of treating primary antibiotics in WWTPs, a straightforward and simplified index could demonstrate optimal efficiency. Unlike the efforts seen in pharmacological treatment,^45^^,^^46^ where concentration-dependent and time-dependent antibiotics are categorized under distinct indices, a universally accepted approach is still lacking among experts. Similarly, a simplified index indicating antibiotic risk could aid in treating antibiotic-containing wastewater in WWTPs, promoting resource efficiency and adherence to regulations.

## Acknowledgments

This work was supported by the 10.13039/501100003725National Research Foundation of Korea (2022R1A2C1007758; RS-2023-00264047) and 10.13039/501100018720Korea Sanhak Foundation (2023-1258-01). SA thanks Architect Priyanjita Adhikari (National Institute of Design, Ahmedabad) for graphical illustrations.

## Author contributions

H.H.L. proposed the outline and guided through the manuscript development. S.A., H.H.L., and D.H.K. jointly organized the manuscript. All of the authors have contributed to the discussion and finalization of the manuscript.

## Declaration of interests

The authors declare no competing interests.
